# Methyl divanillate: redox properties and binding affinity with albumin of an antioxidant and potential NADPH oxidase inhibitor†

**DOI:** 10.1039/c9ra02465d

**Published:** 2019-06-26

**Authors:** Debora Naliati de Vasconcelos, Angélica Nakagawa Lima, Eric Allison Philot, Ana Lígia Scott, Izabelle Amorim Ferreira Boza, Aguinaldo Robinson de Souza, Nelson Henrique Morgon, Valdecir Farias Ximenes

**Affiliations:** Department of Chemistry, Faculty of Sciences, UNESP – São Paulo State University 17033-360 Bauru São Paulo Brazil valdecir.ximenes@unesp.br +55-14-3301-6088; Laboratory of Computational Biology and Bioinformatics, UFABC – Federal University of ABC 09210-580 Santo André SP Brazil; Department of Physical Chemistry, Institute of Chemistry, Campinas State University (UNICAMP) 13083-861 Campinas São Paulo Brazil

## Abstract

Vanillic acid is a widely used food additive (flavouring agent, JECFA number: 959) with many reported beneficial biological effects. The same is true for its ester derivative (methyl vanillate, JECFA number: 159). Based on the increasing evidence that diapocynin, the dimer of apocynin (NADPH oxidase inhibitor), has some improved pharmacological properties compared to its monomer, here the dimer of methyl vanillate (MV), *i.e.*, methyl divanillate (dimer of methyl vanillate, DMV) was synthesized and studied in the context of its redox properties and binding affinity with human serum albumin (HSA). We found that the antioxidant potency of DMV was significantly increased compared to MV. In this regard, the reduction of 2,2-diphenyl-1-picrylhydrazyl (DPPH) free radical by DMV was 30-fold more effective compared to MV. Ferric ion reduction was 4-fold higher and peroxyl radical reduction was 2.7-fold higher. The interaction with HSA was significantly improved (Stern–Vomer constants, 3.8 × 10^5^ mol^−1^ L and 2.3 × 10^4^ mol^−1^ L, for DMV and MV, respectively). The complexation between DMV and HSA was also evidenced by induced circular dichroism (ICD) signal generation in the former due to its fixation in the asymmetric protein pocket. Density-functional calculations (TD-DFT) showed that the ICD spectrum was related to a DMV conformation bearing a dihedral angle of approximately −60°. Similar dihedral angles were obtained in the lowest and most populated DMV cluster poses obtained by molecular docking simulations. The computational studies and experimental displacement studies revealed that DMV binds preferentially at site I. In conclusion, besides being a powerful antioxidant, DMV is also a strong ligand of HSA. This is the first study on the chemical and biophysical properties of DMV, a compound with potential beneficial biological effects.

## Abstract 2

Methyl divanillate, a derivative of the vanillic acid (flavouring agent, JECFA number: 959) with promising beneficial biological effects.

## Graphical abstract

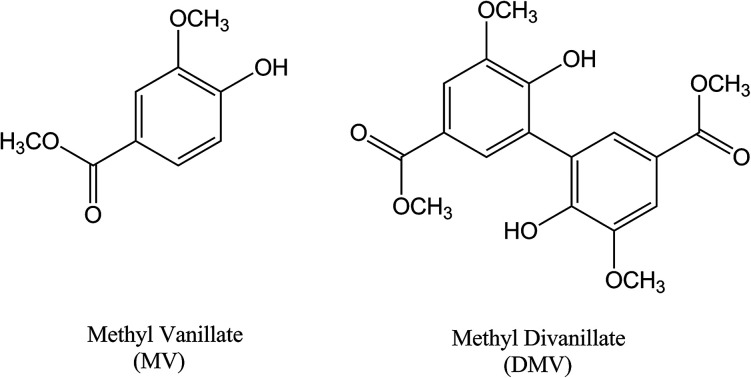

## 1. Introduction

Vanillic acid is a widely used food additive (flavouring agent, JECFA number: 959) with many potential biological applications. The following examples highlight the pharmacologic effects of vanillic acid: ovalbumin-induced asthma mitigation,^1^ neuroprotective agent against cerebrovascular insufficiency states and vascular dementia,^2^ obesity prevention by activating brown adipose tissue thermogenesis,^3^ reactive hyperemia attenuation and improved blood–brain barrier disruption in following transient bilateral common carotid artery occlusion/reperfusion model BCCAO/R^4^ and nephroprotective effect.^5^ From a biological perspective, the ester derivatives of vanillic acid were less studied, but are also promising. This is the case of methyl vanillate, a flavoring agent (JECFA number: 159) originally extracted from *Hovenia dulcis* Thunb.^6^ For instance, methyl vanillate showed a strong inhibitory effect upon antigen-mediated degranulation in rat basophilic leukemia cells.^7^ The effect of methyl vanillate as the most potent inhibitor of macrophage-derived chemokine and thymus activation-regulated chemokine in transformed aneuploid immortal keratinocyte (HaCaT) cells induced by TNF-α and IFN-γ is also relevant.^8^ Methyl vanillate rescued trabecular or cortical femoral bone loss in the ovariectomized mice without inducing any significant weight changes or abnormality in liver tissue when administered orally.^9^ The focus of this work was the dimer of methyl vanillate, *i.e.*, methyl divanillate (DMV). Different from its precursor, DMV does not have a natural origin, and, as far as we known, no biological properties were described to this compound. However, DMV has structural similarity to diapocynin and divanillin, both compounds with well-known important biological effects.^10–12^ Here, methyl divanillate was synthesized and investigated in the context of its redox properties and interaction with serum albumin, the protein responsible for transport of many endogenous and exogenous compounds in the blood circulation.^13^ A comparison was made with methyl vanillate and theoretical studies were performed to elucidate the interaction of DMV with this protein.

## 2. Material and methods

### 2.1 Chemicals and solutions

Human serum albumin (HSA) fatty acid free and essentially globulin free (A3782), bovine serum albumin (BSA) fatty acid free and essentially globulin free (A7030), methyl vanillate, 2,4,6-tri(2-pyridyl)-*S*-triazine (TPTZ), 2,2-diphenyl-1-picrylhydrazyl (DPPH), 2,2′-azobis(2-amidinopropane) hydrochloride (AAPH), 8-hydroxypyrene-1,3,6-trisulfonic acid trisodium salt (pyranine), potassium persulfate, ammonium ferrous sulfate hexahydrate, warfarin, ibuprofen, dansylglycine, and phenylbutazone were purchased from Sigma-Aldrich Chemical Co. (St. Louis, MO, USA). Stock solutions of warfarin, ibuprofen, phenylbutazone, methyl vanillate and its dimer dimethyl divanillate (10 mmol L^−1^) were prepared in dimethyl sulfoxide. From the stock solutions, working solutions (1 mmol L^−1^ or less) were prepared in 50 mmol L^−1^ phosphate buffer at pH 7.0. HSA was dissolved in 50 mmol L^−1^ phosphate buffer at pH 7.0 to give a 1 mmol L^−1^ stock solution.

### 2.2 Dimethyl 6,6′-dihydroxy-5,5′-dimethoxy-[1,1′-biphenyl]-3,3′-dicarboxylate (methyl divanillate, DMV)

DMV was prepared as previously described for the preparation of divanillin with modifications.^14,15^ Methyl vanillate (1.1 g, 6 mmol) was dissolved in 200 mL of hot water and heated until total dissolution. Ammonium ferrous sulfate hexahydrate (118 mg, 0.3 mmol) and potassium persulfate (811 mg, 3.0 mmol) were added, the heating was kept for additional three minutes, turned off, and stirred for additional 30 min. The precipitated product was hot-filtered and washed with 500 mL of hot water and with 500 mL of cold water. The product was dried in an oven at 60 °C for 24 hour and then in a vacuum desiccator over phosphorus pentoxide for additional 24 hour, yielding 0.78 g (72%) of a yellow pale solid. The purity (>96%) was confirmed by HPLC analysis (Jasco, Tokyo, Japan) (Fig. S3†). The analyses were carried on a Luna C18 reversed-phase column (250 × 4.6 mm, 5 μm) using solvent A (aqueous formic acid 0.1%) as a mobile phase and solvent B (formic acid 0.1% in acetonitrile). The gradient was: solvent A 80% to 20% in 20 min. The flow rate was 1 mL min^−1^. NMR spectra were obtained using DMSO-D_6_ as solvent and internal reference for ^1^H and ^13^C (Bruker DRX 400 spectrometer, MA, USA): ^1^H NMR (400 MHz) *δ* (ppm): 9.53 (s, 2OH), 7.47 (d, 2CH-Ar, *J* = 2.0 Hz), 7.44 (d, 2CH-Ar, *J* = 2.0 Hz), 3.91 (s, 2 OCH3), 3.81 (s, 2 OCH3). ^13^C NMR (100 MHz, DMSO-D_6_) d (ppm): 166.1 (2C=O), 148.8 (2C), 147.4 (2C), 125.4 (2CH), 124.3 (2C), 119.5 (2C), 110.9 (2CH), 56.0 (2OCH3), 51.8 (2OCH3) (Fig. S1 and S2†). The UV-Vis and FTIR spectra are also available in Fig. S3.†

### 2.3 DPPH scavenging assay

MV and DMV were incubated for 30 min with 100 mmol L^−1^ DPPH in ethyl alcohol in the dark. The scavenging activity was evaluated spectrophotometrically at 517 nm using the absorbance of unreacted DPPH radical as a control and calculated as: [(absorbance of control − absorbance of sample)/(absorbance of control)] × 100.^16^

### 2.4 Ferric reducing antioxidant power (FRAP) assay

These studies were performed as previously described with modifications.^17^ The FRAP reagent was prepared as follow: 1 mL TPTZ (10 mmol L^−1^ dissolved in 40 mmol L^−1^ HCl), 1 mL FeCl_3_ (20 mmol L^−1^ dissolved in water) and 10 mL sodium acetate buffer, 300 mmol L^−1^, pH 3.6. Various concentrations of the tested compounds (10 μL) were incubated with 290 μL of FRAP reagent for 30 minutes in the dark at room temperature. The absorbance was measured at 593 nm using a mixture comprised of 10 μL of 50 mmol L^−1^ phosphate buffer, pH 7.0, and 290 μL FRAP as the blank. The relative antioxidant efficacy was evaluated by the analytical curve slope, which correlates the relative complexed Fe^2+^ concentration with the antioxidant concentration.

### 2.5 Reactivity with peroxyl radical: pyranine assay

Pyranine (10 μmol L^−1^) was incubated with 20 mM AAPH in 50 mmol L^−1^ phosphate buffer, pH 7.0 at 37 °C in the absence (control) or presence of the studied compounds in the wells of a black microplate. The reactions were conducted by 2 hours and the fluorescence bleaching of pyranine was monitored at excitation of 460/510 nm, excitation and emission, respectively in a Spectramax M2 microplate reader (Molecular Devices, Sunnyvale, CA). The final reaction volume was 250 μL. The ratio between the area under the curve in the absence or presence of the studied compounds were measured and plotted against concentration. The slopes of the analytical curves obtained by plotting the area under the curve of the sample divided by area under the curve of the control (AUCs/AUCc) against concentration of the studied compounds were used as analytical parameter.^18^

### 2.6 Protein binding assays

The interactions of MV and DMV with HSA and BSA were evaluated by intrinsic fluorescence quenching of the proteins. HSA and BSA (2.5 μmol L^−1^) were titrated with the studied compounds in 50 mmol L^−1^ phosphate buffer, pH 7.0, at different temperatures. After each addition, the protein/ligand mixtures were incubated for 2 min before the fluorescence measurements. The fluorescent intensities were corrected for the inner filter effect caused by attenuation of the excitation and emission signals provoked by the absorption of divanillin using eqn (1).^19^

1 *F*_corr_ = *F*_obs_ × 10^(Ab_ex_+Ab_em_)/2^

In this equation *F*_corr_ and *F*_obs_ are the corrected and observed fluorescence intensities, respectively. Ab_ex_ and Ab_em_ are the absorptions of the mixture at excitation (295 nm) and emission wavelengths (343 nm), respectively. The absorbance and fluorescence spectra were measured using a PerkinElmer Lambda 35 UV-visible spectrophotometer and PerkinElmer LS 55 spectrofluorimeter, respectively (Shelton, CT, USA). The fluorescence experiments were performed with the following settings: excitation at 295 nm and emission in the range 310 nm to 450 nm. The slit widths were 10 nm for both excitation and emission wavelengths. The experiments were performed using a 3 mL quartz cuvette with a 10 mm path length and magnetically stirred during the measurements. The linear and non-linear fittings for Stern–Volmer and association constants were obtained using the GraphPad Prism version 5.00 for Windows (GraphPad Software, San Diego California USA).

### 2.7 Induced circular dichroism (ICD) experiments

The complexation between DMV and HSA was studied by the induction of chirality. The reaction medium was composed of HSA (30 μmol L^−1^) in the absence or presence of 30 μmol L^−1^ dimethyl divanillate in 50 mmol L^−1^ phosphate buffer, pH 7.0. The ICD studies were performed in a Jasco J-815 spectropolarimeter (Jasco, Japan) equipped with a thermostatically controlled cell holder. The spectra were obtained with 1 nm step resolution, response time of 1 s and scanning speed of 50 nm min^−1^. A 3 mL quartz cuvette with a 10 mm path length and a magnetic stirrer were used for the measurements in the near-UV-CD range. The baseline (50 mmol L^−1^ phosphate buffer) was subtracted from all measurement.

### 2.8 Binding site determination

The characterization of the binding site of DMV in HSA was performed by displacement of the fluorescent site markers warfarin and dansylglycine. For warfarin, the spectrofluorimeter was adjusted to excitation at 310 nm and emission in the range 330–450 nm. The experiments were performed by the addition of varying amounts of dimethyl divanillate, ibuprofen or phenylbutazone in 5 μmol L^−1^ HSA and 5 μmol L^−1^ warfarin in 50 mmol L^−1^ phosphate buffer at pH 7.0.^15^ For dansylglycine, the spectrofluorimeter was adjusted to excitation at 360 nm and emission in the range 400–600 nm. The experiments were performed by the addition of varying amounts of the dimethyl divanillate, ibuprofen or phenylbutazone to a mixture of 5 μmol L^−1^ BSA and 10 μmol L^−1^ dansylglycine in 50 mmol L^−1^ phosphate buffer at pH 7.0. The mixtures were incubated for 2 min at 25 °C before the measurements.^20^

### 2.9 Density functional theory (TD-DFT) studies: low energy conformation and simulation of ECD

The most stable conformation adopted by DMV was calculated using the B3LYP/6-311++G(2d,p) hybrid exchange–correlation functional. All computer simulations were done in the GridUnesp supercomputer facilities, which is composed of 256 SUN X4150 servers with 2048 cores (Intel Xeon 2.83 GHZ), with 4096 GB of RAM memory (2 GB per core) and an infiniband 4X DDR (20 Gbps) connection. The storage capacity of these system is 36 TB through DAS optical fibre (StorageTek 6140) and 96 TB at four SUN X4500 servers. The Gaussian09 suite of programs was employed to obtain the geometric and energy parameters for DMV.^21^ The simulation of the electronic circular dichroism (ECD) spectrum was carried out using the CAM-B3LYP, a hybrid exchange–correlation functional that combines the hybrid qualities of B3LYP and the long-range correction, and 6-311++G(2df,p) basis set. The solvent was modelled using the Polarizable Continuum Medium (PCM) using the integral equation formalism variant (IEFPCM).

### 2.10 Docking simulations

Molecular docking was applied in order to investigate the binding between HSA with DMV. Since there are two main binding sites for drugs described in the literature, Sudlow's drug site 1 and 2, the docked regions that comprise each binding site were studied separately. AutoDock^22^ (version 4.2.6) and AutoDock Tools^22,23^ (version 1.5.7rc1) were employed for docking simulations and input file preparation for the simulation, respectively. Ligand–protein interaction was analyzed with the aid of the BINANA program^24^ with default parameters. The figures presented in the paper were made with PyMOL (Version 1.7.0.0 – The PyMOL Molecular Graphics System, Version 1.7.0.0 Schrödinger, LLC). The apo structure, PDB ID: 1AO6 (ref. 25) and HSA-propofol complex, PDB ID: 1E7A^26^ were used for simulations. All water molecules were removed. Polar hydrogen atoms and Gasteiger charges were added to the ligand and protein using AutoDock Tools. The docking grid spacing was set to 0.375 Å, centered on 24.37 (*x*), 28.65 (*y*) and 29.29 (*z*) for site I and 13.73 (*x*), 26.00 (*y*) and 19.00 (*z*) for site II. Box sizes in *X*, *Y* and *Z* directions were 32.0, 32.58 and 34.53 for site I, and 13.73, 26.0 and 19.0 for site II, respectively. The docking parameters used were: Lamarckian Genetic Algorithm (LGA), with a population size of 150, and maximum number of generations and energy evaluations equal to 27 000 and 25 000 000, respectively. Other parameters were set as default values. The protein was kept rigid and the ligand flexible during molecular docking. The total number of runs were set at 100 in order to generate different conformations. Each conformation was visually inspected, and the selected poses were analyzed with the BINANA software after docking calculation.

## 3. Results and discussion

### 3.1 Synthesis and redox properties

**Fig. 1:**
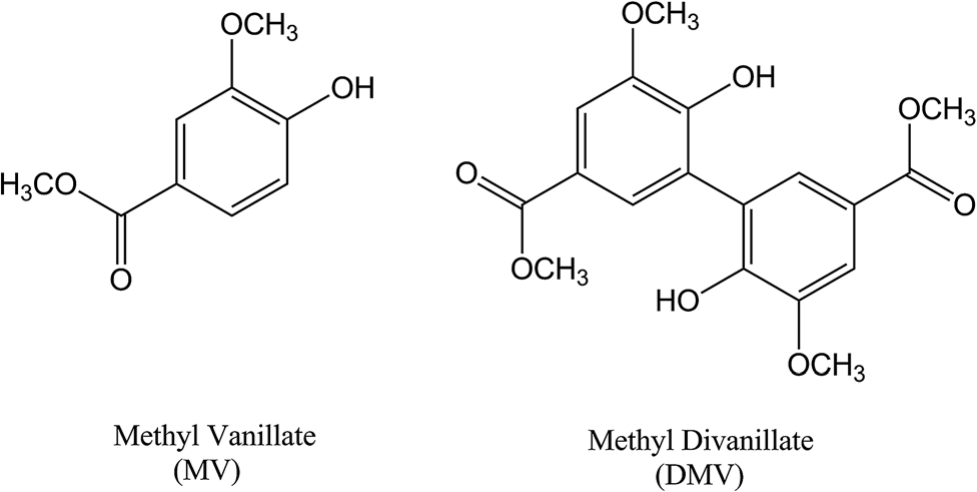
Molecular structures of the studied compounds.

The dimerization of MV to generate DMV was performed through ferrous sulphate catalyzed oxidation using sodium persulphate as the oxidizing agent.^14,15^ Its purity (>98%) was evaluated by HPLC and its identity by NMR (Fig. S1–S3†). Fig. 1 shows the molecular structures of MV and DMV.

**Fig. 2:**
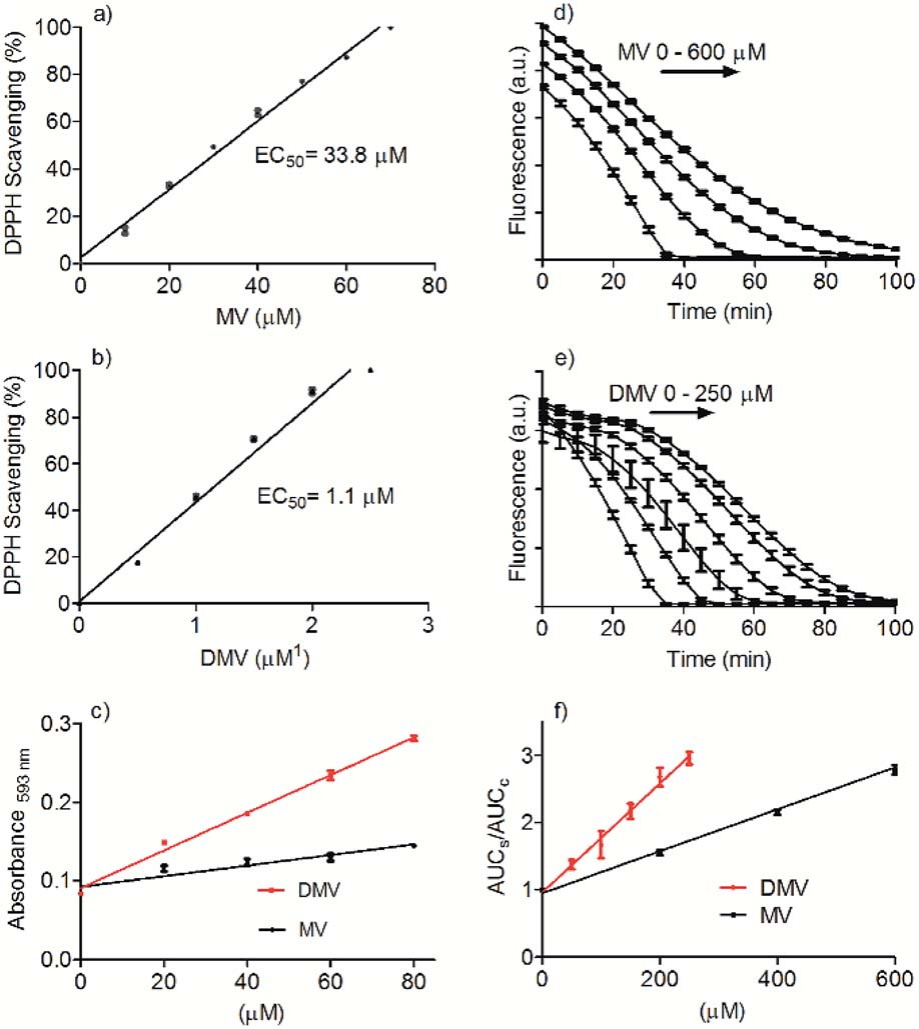
Comparative evaluation of antioxidant activity of MV and DMV. (a and b) Reduction of DPPH free radical. (c) Reduction of Fe^2+^ (FRAP assay). The slopes were MV 6.7 × 10^−4^ and DMV 2.4 × 10^−3^. (d–f) Reactivity with peroxyl radical. The slopes were MV 3.0 × 10^−3^ and DMV 8.1 × 10^−3^. The results are mean and SD of triplicates.

The DMV antioxidant properties were significantly improved compared to MV. Fig. 2 shows the results obtained in the antioxidant activity evaluation methods based on the reduction of DPPH free radical and reduction of ferric ion (FRAP). With respect to reduction of DPPH, DMV was around 30-fold more potent compared to MV, EC50 1.1 μmol L^−1^ and 33.8 μmol L^−1^, respectively (Fig. 2a and b). Regarding their capacities as reducing agent of ferric ion, the efficiency of DMV was 4-fold higher compared to MV (slopes 2.4 × 10^−3^*versus* 6.7 × 10^−4^, respectively) (Fig. 2c). The studied compounds were also able to reduce peroxyl radical generated by temperature-induced degradation of AAPH. This experiment is based on fluorescence decay of pyranine when submitted to oxidation by peroxyl radicals.^27^ In this methodology, the effectivity of an antioxidant is based on its competition with pyranine by peroxyl radical. The results depicted in Fig. 2d–f show that DMV was 2.7-fold more potent than MV (slopes 3.0 × 10^−3^*versus* 8.1 × 10^−3^).

All together, these results are consistent with the increased electron delocalization promoted by the linkage of the two aromatic rings and the consequent decrease in the oxidation potential of DMV. Corroborant with these findings, dimers as diferulic acid and diapocynin were also reported as better antioxidants compared to their monomer precursors.^11,28^ It is worthy of note that MV is a methoxy-substituted catechol, a family of compound for which the potential therapeutic application is continuously increasing. The best example of this assertion is apocynin (acetovanillone). Indeed, since the first demonstration of its inhibitory action upon the multienzymatic complex NADPH-oxidase in neutrophils in 1994,^29^ more than 2000 papers have been published confirming its healthy beneficial properties in numerous experimental models. Other examples of methoxy-substituted catechols are vanillin and vanillic acid, which besides widely used as flavouring agents, have numerous pharmacological properties.^7,10,30–32^ Homodimers of these methoxy-substituted catechols also have biological effects and, sometimes, superior to their precursors, as have been demonstrated to diapocynin, and divanillin.^10,11,33,34^ Usually, the antioxidative efficacy of these dimers is increased. Here, this was exactly what we found by comparing MV and DMV. In short, the facility by which DMV can be synthesized, the improved antioxidant activity and the similarity with diapocynin and divanillin suggest that DMV should be submitted to further cell-based and *in vivo* studies. In this regard, the cytotoxicity of DMV was evaluated and compared with its precursor MV. This task was accomplished using peripheral blood neutrophil as the target cell and the MTT assay, which assesses cell metabolic activity, as analytical procedure to evaluate the cell viability. Neutrophil was chosen since this cell type is a common source of NADPH-oxidase.^35^ Table S1† shows the cell viability in the presence of DMV and MV. This result is a confirmation that DMV is as safe as MV and comparable with the similar compounds diapocynin and divanillin, which were noncytotoxic HepG2 cells at same concentration.^10^

### 3.2 Binding with albumin

**Fig. 3:**
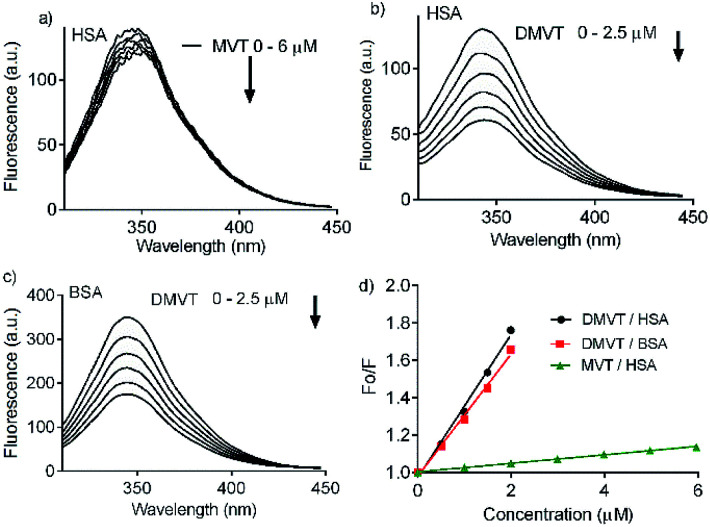
Intrinsic fluorescence quenching of albumin provoked by complexation with MV and DMV. (a) HSA 2.5 μmol L^−1^ and MV 0–6 μmol L^−1^. (b) HSA 2.5 μmol L^−1^ and DMV 0–2.5 μmol L^−1^. (c) BSA 2.5 μmol L^−1^ and DMV 0–2.5 μmol L^−1^. (d) Stern–Volmer plots.

Besides a better antioxidant, DMV was also significantly more effective as albumin ligand compared to its precursor. This feature is shown in Fig. 3 where the protein intrinsic fluorescence quenching was measured as a function of the ligand concentration. For illustration, while 6 μmol L^−1^ of MV resulted in 16% fluorescence depletion (Fig. 3a), only 2.5 μmol L^−1^ of DMV provoked 53% (Fig. 3b). It is worthy of note that the concentration of protein (2.5 μmol L^−1^) and ligands (0–2.5 or 0–6.0 μmol L^−1^) were chose to avoid the non-specific quenching effect due to inner-filter effect caused by the light absorption. However, the subtle inner-filter effect was still corrected using eqn (1) (Material and methods). The Stern–Volmer (*K*_sv_) constants were calculated using eqn (2).2
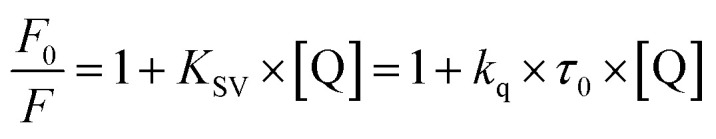
In this equation *F*_0_ and *F* are the fluorescence intensity in the absence and presence of the quenchers (MV or DMV). The obtained Stern–Volmer constants revealed that the interaction of DMV with HSA was 16-fold more efficient compared to MV (3.8 × 10^5^ mol^−1^ L and 2.3 × 10^4^ mol^−1^ L, respectively) (Fig. 3d). The magnitude of these constants is consistent with the formation of complex between the proteins and ligands. This conclusion can be reached assuming the average lifetime of excited tryptophan residues in the albumin (*τ*_0_) as ∼6 ns.^36^ From this assumption, the bimolecular quenching constant (*k*_q_) resulted in 6.3 × 10^13^ mol^−1^ s^−1^ L for DMV and 2.5 × 10^12^ mol^−1^ s^−1^ L for MV. These values are significantly higher compared to the maximum collision quenching constant, 2 × 10^10^ mol^−1^ s^−1^ L,^37^ hence a static process (*i.e.*, a complexation in ground state) can be attributed as responsible by the fluorescence quenching. The high affinity of DMV as ligand was not exclusive to HSA, but also to BSA, as depicted in Fig. 3c and d.

**Fig. 4:**
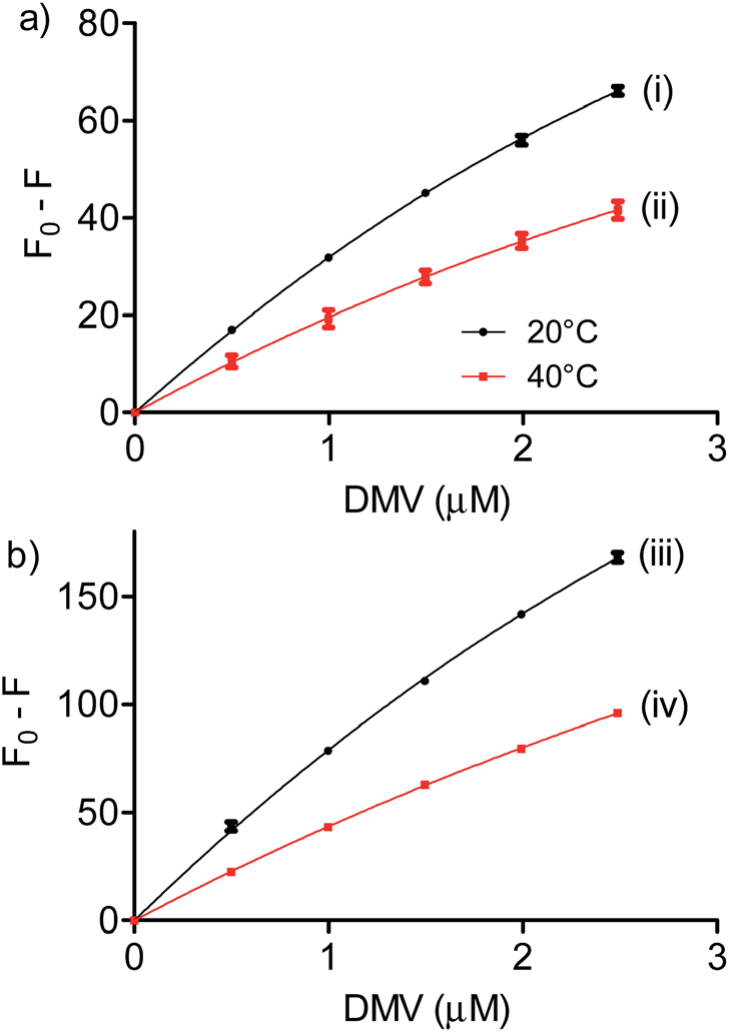
Determination of association constants between albumins and DMV. (a) HSA and (b) BSA. The results are means and SD of triplicates (*R*^2^, 0.9991 (i), 0.9901 (ii), 0.9992 (iii) and 0.9997 (iv)).

Once found that the quenching phenomenon was linked to the complexation between HSA and DMV, the results were fitted to eqn (3) and the binding constants determined.^38^3
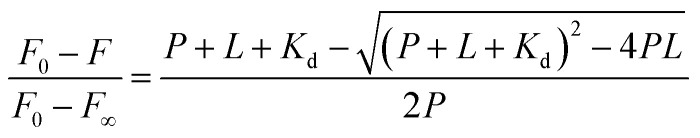
Besides the parameters already defined above, *P* is the fixed protein concentration; *L* is the concentration of ligand (DMV); *F*_∞_ is the fluorescence of the protein at infinite concentration of DMV; *K*_d_ is the dissociation constant. Here, a 1 : 1 stoichiometry was assumed and the association constant (*K*_a_) was calculated as 1/*K*_d_. Fig. 4 shows the non-linear fitting of *F*_0_ − *F* as a function of DMV concentration. It is worthy of note that *F*_∞_ was obtained as an equation parameter and not experimentally obtained. Table 1 shows the association constants obtained at 20 °C and 40 °C for both proteins. The magnitudes of the association constants (∼10^5^ mol^−1^ L) are compatible with values usually observed for drugs well-established as efficient ligands of albumin as warfarin, ibuprofen, diazepam and dansylated amino acids.^39–42^ The high binding efficacy of biphenyls compared to their monomers is not exclusive of DMV; indeed, similar effect was described to diapocynin (5.1 × 10^5^ mol^−1^ L) compared to apocynin (2.2 × 10^4^ mol^−1^ L).^43,44^ Other example of strong interaction (∼10^6^ mol^−1^ L) was related to brominated diphenyl esters.^45^ Probably, the increased hydrophobicity of these compounds compared to their monomers precursors is behind of the augmented binding efficacy. This dependence has also been demonstrated to bile salts derivatives.^46^

**Table 1:** Association constants and thermodynamic parameters for the complexation between albumins and methyl divanillate

	*T* (K)	*K*_a_ (10^5^ mol^−1^ L)	Δ*H*° (kJ mol^−1^)	Δ*G*° (kJ mol^−1^)	Δ*S*° (J mol^−1^ K^−1^)
BSA	293	3.5	−24.9	−31.1	20.9
	313	1.8		−31.5	
HSA	293	7.6	−29.8	−32.9	10.8
	313	3.5		−33.2	

We also found that the association constants decreased at higher temperature, which is consistent with the formation of a complex between protein and ligand and reinforce the conclusion that a static process is responsible by the observed fluorescence quenching.^37^ From these results and the application of eqn (4)–(6), the thermodynamic parameters were obtained (Table 1). These values are consistent with the spontaneity of the interaction (negative Δ*G*) and indicated that favorable enthalpic and entropic effects are behind of the interactions.^47^ Finally, it is worthy of note that the interaction of DMV with HSA did not provoked any alteration in the secondary structure of the protein, as evaluated by Far-UV circular dichroism (Fig. S5†).4
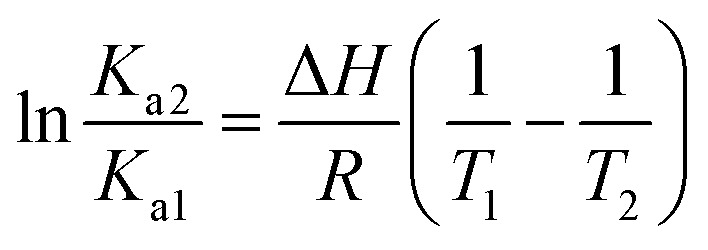

5 Δ*G* = −*RT* ln

6
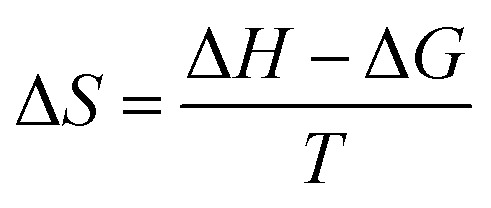


### 3.3 Experimental ICD and theoretical studies

**Fig. 5:**
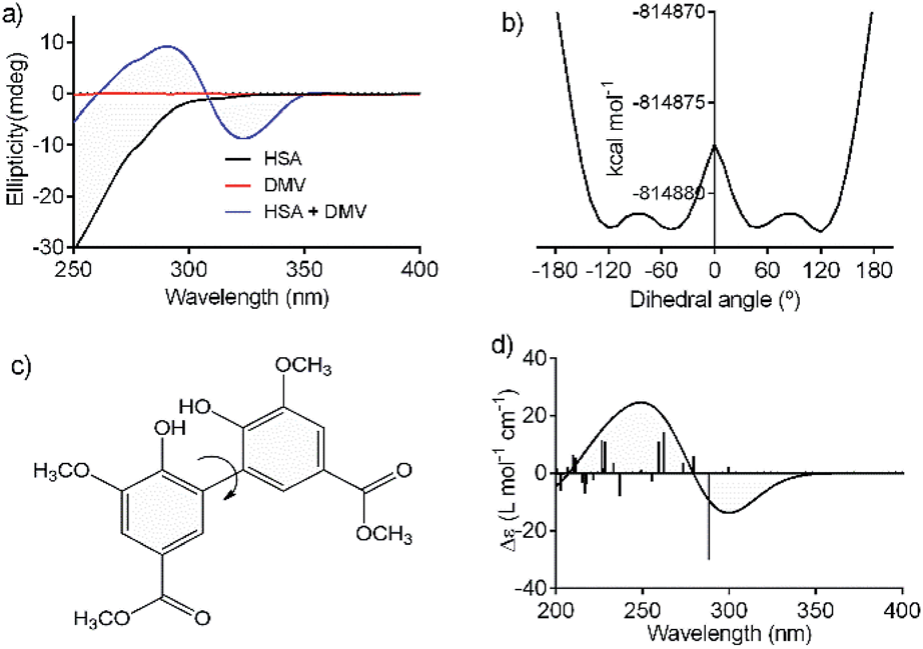
Induction of chirality in DMV by complexation with HSA. (a) Experimental ICD spectra of DMV in the absence and presence of HSA. (b) TD-DFT calculated total energy of the conformations of DMV generated by rotation of the dihedral angle between the aromatic rings (c). (d) TD-DFT simulated ECD spectrum to the −60° conformer.

An additional evidence of the complexation between DMV and HSA was obtained by circular dichroism. Fig. 5a shows the DMV CD spectrum in the absence and presence of HSA. The negative band with maximum centered at 324 nm and the positive one at 290 nm were only observed in the presence of HSA. This is a typical case of induction of chirality in an achiral guest (ligand) due to its interaction with a chiral host (protein), *i.e.*, a case of induced circular dichroism (ICD).^48^ Aiming to explain the origin of the ICD signal, DMV was submitted to *ab initio* calculations. It was assumed that the induced optical activity could be related to a non-planar conformation of DMV inside the protein and the free rotation around its biphenyl system should be impeded due to the restriction of movement in the cavity. Hence, the initial approach was to obtain the total energy for each conformation of DMV. This task was performed using the SCAN keyword available at the Gaussian suite of programs. The quantum chemical formalism used to search for the most stable conformations adopted by DMV in the gas phase was carried out at B3LYP/6-31G(d) high level of theory. Fig. 5b shows the minimum energy conformations obtained by scanning the dihedral angle around the bond that connect the aromatic rings (Fig. 5c). The minima were observed at ∼60° and equivalent angles. The energy barrier for the conversion of these conformation was ∼1 kcal mol^−1^. This result explains the absence of optical activity for DMV free in solution, since it has been demonstrated that a minimum of 93 kcal mol^−1^ must be the difference for a biphenyl system to exist as stable enantiomers at 300 K.^49^ The next step was to simulate the CD spectra of the minimum energy conformations (Fig. 5d). The best result was obtained for the conformer with dihedral angle around −60°, which generated CD spectrum with good similarity to the experimental one (Fig. 5a). The presence of similar conformer of DMV inside that protein was confirmed by docking simulation as will be demonstrated in the next section. In short, the existence an optically active chiral conformation of DMV is an indication that intermolecular forces involved in its fixation inside the protein impeded the free rotation of the biphenyl ring system. In other words, it is an additional confirmation of the binding of DMV in HSA.

### 3.4 Determination of binding sites

**Fig. 6:**
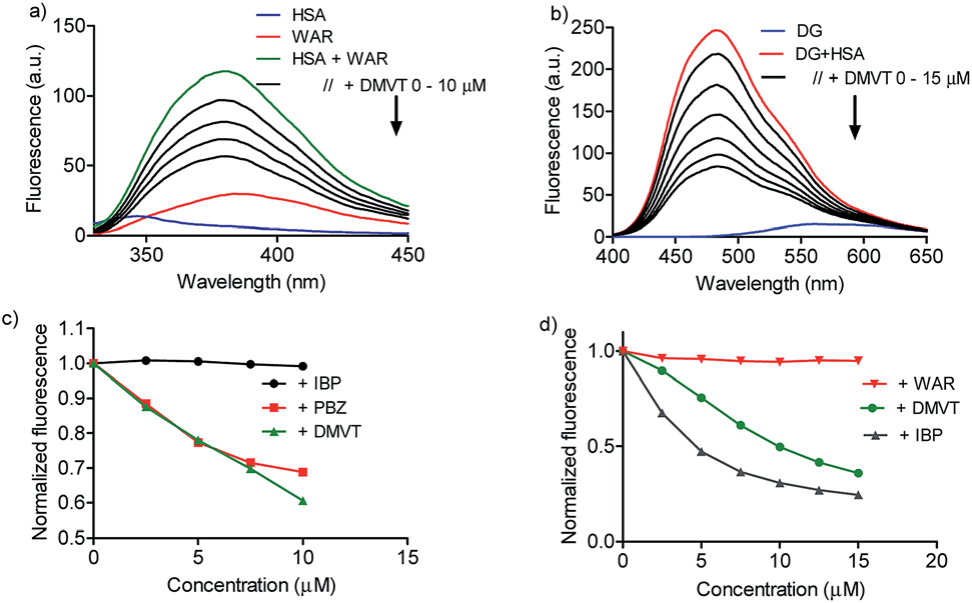
Displacement of warfarin (site I) and dansylglycine (site II) by DMV. (a and b) HSA 5 μmol L^−1^, warfarin (WAR) 5 μmol L^−1^ and increasing concentration of DMV, ibuprofen (IBP) and phenylbutazone (PBZ). (c and d) HSA 5 μmol L^−1^, dansylglycine (DG) μmol L^−1^ and increasing concentration of DMV, IBP and WAR.

Once demonstrated that DMV was able to bind in HSA, the next step was to search for putative preferential binding sites. To do so, warfarin was used as a marker of site I. This drug, besides a well-established site I ligand, has its intrinsic fluorescence increased when bound to albumin.^50^ The results depicted in Fig. 6a highlight this photophysical feature and shows that the addition of DMV decreased the fluorescence of warfarin. This result can be interpreted as the displacement of warfarin from its binding site. To reinforce this point of view, it is also shown the displacement provoked by phenylbutazone, site I ligand, and ibuprofen, site II ligand.^39,40,42^ How can be seen, phenylbutazone, but not ibuprofen, was able to displace warfarin, and its efficacy was similar to DMV.

To search for the affinity of DMV to site II, dansylglycine was used as fluorescent markers. Dansylglycine also have its fluorescence significantly increased by complexation at site II and its displacement is a tool to search for site II ligands.^20^ The results depicted in Fig. 6c and d show that DMV was also able to displace dansylglycine, even though with less efficiency compared to ibuprofen. Altogether, these results are indicative that DMV was able to bind in both binding site of HSA, but with a preference for site I.

**Fig. 7:**
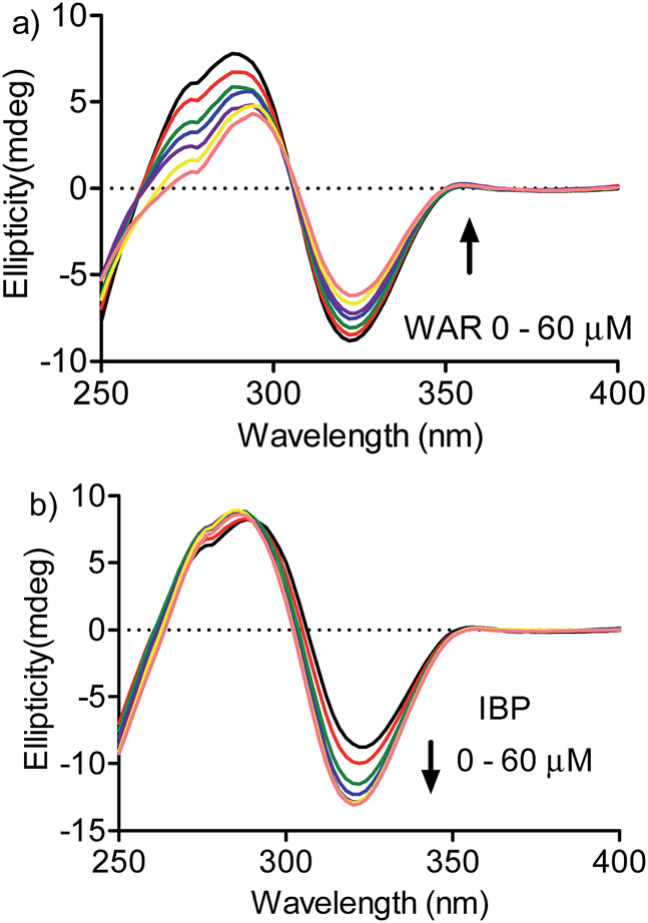
Displacement of DMV by warfarin and ibuprofen monitored by ICD. HSA 30 μmol L^−1^, DMV 30 μmol L^−1^ and increasing concentration of (a) WAR and (b) IBP.

A confirmation of the preference for site I was obtained by measuring the effect of warfarin and ibuprofen in the DMV ICD spectrum. Fig. 7a shows that warfarin was able to provoke a decrease in the ICD intensity, which suggest the partial displacement of DMV. Contrarily, the addition of ibuprofen provoked an increase in the ICD signal of DMV and also a small shift for lower wavelength. This result can be interpreted as an allosteric-like effect, as have been demonstrated for ethacrynic acid, which increased the ICD signal of bilirubin.^48^ In short, it suggests that the binding of ibuprofen at site II could provoke a small alteration in the tertiary structure of the protein with reflex in the site I and its interaction DMV.

### 3.5 Docking studies

The experimental results indicated that DMV is a strong HSA ligand. Hence, molecular docking analysis were performed in order to elucidate the features of the interaction as well as to validate these findings. Two crystallographic structures were selected: 1AO6,^25^ an apo structure; and 1E7A,^51^ which contains two propofol molecules in the crystal – one at subdomain IIIA and other at subdomain IIIB. Displacement assays revealed that, even though a preference was observed to site I, DMV was able to bind in both sites. Hence, the docking simulations were independently performed for each of these regions. A box was defined where the flexible ligand was free to accommodate in the rigid protein for each binding site. One hundred experiments were independently performed, and the poses were clustered following the AutoDock (RMSD < 2 Å) default criteria. The lowest energies and number of poses for each crystal structure cluster (1AO6 and 1E7A) are depicted in the histograms (Fig. S5a, S5b, S6a and S6b†). Table 2 presents, separately, the energy for the most populated and lowest energy clusters. Assuming that DMV can be internally bound in each site, the poses were also analyzed under this criterium and highlighted in grey (Table 2).

**Table 2:** Binding energy for the most populous and minimum energy clusters of DMV in HSA. In grey is highlighted the clusters located internally in the binding sites

		Cluster (*n*° poses)	Binding energy (kcal mol^−1^)
Site I	1AO6	Cl-1 (60)	−8.33
	1E7A	Cl-1 (10)	−7.01
		Cl-8 (29)	−6.05
Site II	1AO6	Cl-1 (7)	−6.18
		Cl-2 (34)	−6.03
	1E7A	Cl-1 (42)	−6.82

#### Interactions at site I

**Fig. 8:**
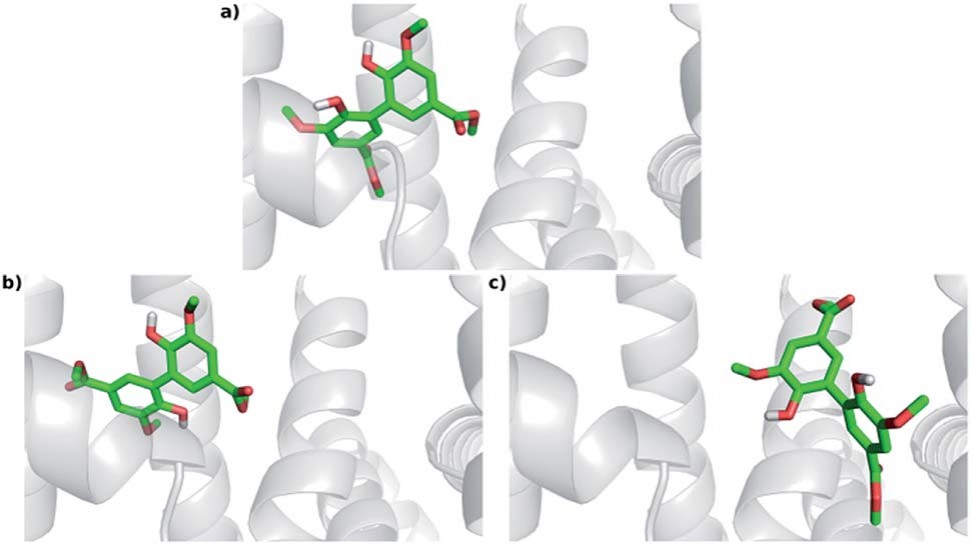
Docking poses for DMV at site I. (a) PDB 1AO6 – lowest energy and most populated cluster (Cl-1); (b) PDB 1E7A – lowest energy cluster (Cl-1); (c) PDB 1E7A – most populated cluster (Cl-8).

The lowest energy structures that belong to both 1A06's cluster 1 (Cl-1: lowest energy and most populated) and 1E7A's cluster 1 (Cl-1: lowest energy cluster) are located within site I. On the other hand, the lowest energy structures that belong to 1EA7's cluster 8 (Cl-8: most populated) are located at site I's entry (Fig. 8).

**Fig. 9:**
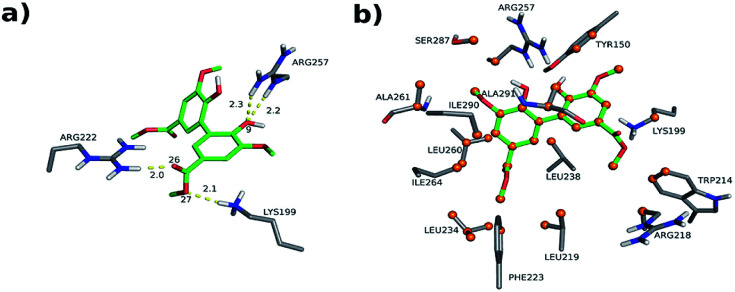
Intermolecular interactions of DMV at site I of HSA (PDB 1AO6). The results represent the most populated and lower energy cluster. (a) Hydrogen bonds; (b) hydrophobic contacts.

The interactions between DMV and the amino acid residues at site I that take place in each of these poses were analyzed by the Binana software, and are depicted in Fig. 9, S7, S8, Tables S2 and S3.† The interaction details are as follows:

- PDB 1AO6 Cl-1: four hydrogen bonds between residues Lys 199, Arg 222 and Arg 257: (i) one between DMV's oxygen (O26) and Arg 222's hydrogen (1HH2) at distance of 2.0 Å; (ii) two between DMV's oxygen O9 and Arg 257's hydrogens (HE and 2HH2 at a distance of 2.2 Å and 2.3 Å, respectively); and (iii) one between DMV's oxygen (O27) and Lys 199's hydrogen (HZ1) at a distance of 2.1 Å (Fig. 9a). Forty-four hydrophobic contacts with several residues (Fig. 9b).

- PDB 1E7A Cl-1: five hydrogen bonds between residues Lys 199, Arg 222 and Arg 257: (i) one between DMV's oxygen (O1) and Lys 199's hydrogen (1HZ2) at a distance of 2.5 Å; (ii) one between DMV's oxygen (O23) and Arg 257's hydrogen (HE) at a distance of 2.2 Å; (iii) two between DMV's oxygen (O26) and Lys 199's and Arg 222's hydrogens (HZ1 an 1HH1) at a distance of 2.9 Å and 1.9 Å, respectively; (iv) one between DMV's oxygen (O27) and Arg 222's hydrogen at a distance of 1.7 Å (Fig. S7a†). Thirty-three hydrophobic contacts with several residues (Fig. S7b†).

- PDB 1E7A-Cl8: four hydrogen bonds between residues Lys 195, Lys 199, Arg 218 and Arg 222 (Fig. S8†): (i) two between DMV's oxygen (O1) and Lys 199's and Arg 222's hydrogens (HZ1 and 1HH2) at distance of 2.2 Å and 2.8 Å, respectively; (ii) one between DMV's oxygen (O9) and Arg 218's hydrogen (HE) at a distance of 2.2 Å; (iii) one between DMV's oxygen (O17) and Lys 195's hydrogen (HZ3) at a distance of 2.3 Å (Fig. S7a†). Thirty-five hydrophobic contacts with several residues (Fig. S8b†). One cation-π with Lys 199 (numbered ring of 3 to 8 at Fig. S8c†). Two π stacking (T-shaped) between the Trp 214 rings and a DMV ring (numbered 3–8, Fig. S8d†).

#### Interactions at site II

**Fig. 10:**
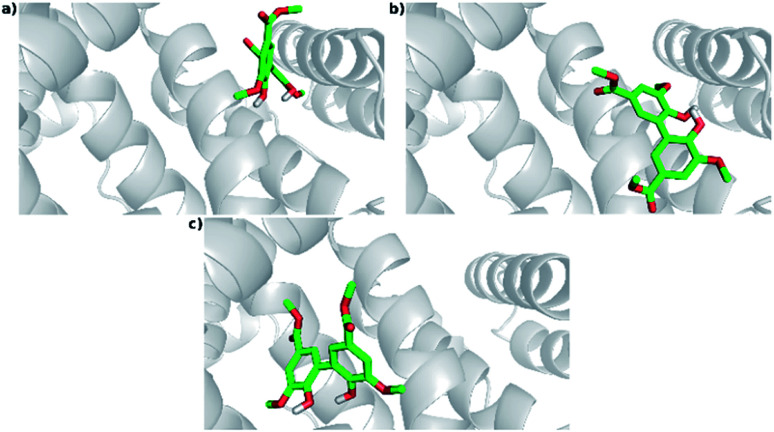
Poses for DMV at site II. (a) PDB 1AO6 – lowest energy cluster (Cl-1); (b) PDB 1AO6 – most populated cluster (Cl-2); (c) PDB 1E7A – most populated and lower energy cluster (Cl-1).

The lowest energy structure of 1EA7's cluster 1 (Cl-1: lowest energy and most populated cluster) is located within site II, whereas 1AO6's cluster 1 (Cl-1: lowest energy) and 2 (Cl-2: most populated cluster) are external (Fig. 10).

**Fig. 11:**
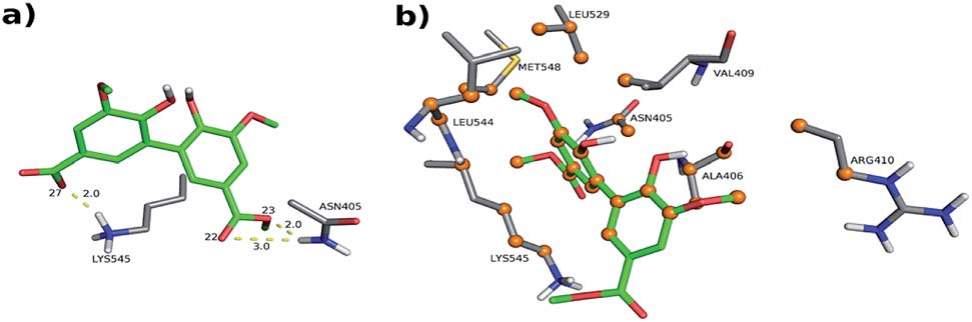
Intermolecular interactions of DMV at site II of HSA (PDB 1AO6). The results represent the lower energy cluster (Cl-1). (a) Hydrogen bonds; (b) hydrophobic contacts.

The interactions details at site II are as follows (depicted in Fig. 11, S9, S10, Tables S2 and S3†):

- PDB 1AO6 Cl-1: three hydrogen bonds between residues Asn 405 and Lys 545: (i) one between DMV's oxygen (O22) and Asn 405's hydrogen (2DHD2) at a distance of 3.0 Å; (ii) one between DMV's oxygen (O23) and Asn 405's hydrogen (2HD2) at a distance of 2.0 Å; (iii) one between DMV's oxygen (O27) and Lys 545's hydrogen (HZ3) at a distance of 2.0 Å (Fig. 11a). Forty-seven hydrophobic contacts with several residues (Fig. 11b).

- PDB 1AO6 Cl-2, site II: three hydrogen bonds with the residues Arg 410 and Lys 413 (Fig. S10†): (i) one between DMV's oxygen (O2) and Lys 413's hydrogen (HZ2) at a distance of 2.2 Å; (ii) one between the DMV's oxygen (O17) and Lys 413's hydrogen (HZ3) at a distance of 2.2 Å; (iii) one between DMV's oxygen (O22) and Arg 410's hydrogen (1HH1) at a distance of 2.2 Å (Fig. S10a†). Thirty-three hydrophobic contacts with several residues (Fig. S10b†).

- PDB 1E7A Cl-1: two hydrogen bonds with the residues Asn 391 and Arg 410: (i) one between DMV's oxygen (O9) and Arg 410's hydrogen (HE) at a distance of 2.8 Å; (ii) one between DMV's oxygen (O27) and Asn 391's the hydrogen (2HD2) at a distance of 1.9 Å (Fig. S11a†). Fifty-five hydrophobic contacts with several residues (Fig. S11b†). One cation-π with Arg 410 (numbered ring of 3 to 8 at Fig. S11c†). One π stacking (T-shaped) between the Tyr 411 rings and a ring (numbered 11–16, Fig. S11d†) of DMV.

In summary, it was possible to observe that internal DMV poses in site I were deeply buried in a hydrophobic region, accomplishing several hydrophobic contacts with nearby residues. Site I has two clusters of polar residues (Tyr 150, His 242, Arg 257; and Lys 195, Lys 199, Arg 218, Arg 222). In these DMV poses, the compound was able to make hydrogen bonds with residues of the both polar groups. For the site II, the internal DMV pose (obtained for the 1E7A structure) were partially buried in the cavity, with one ring buried and the other partially exposed to the solvent. This positioning is consistent with the characteristic of site II, in which that hydrophobic cavity is smaller than the one found in site I, besides being exposed to the solvent. This hydrophobic feature enables the DMV to establish several hydrophobic contacts with nearby radius. Another feature of site II is the presence of one polar patch residue composed by Arg 410, Tyr 411, Lys 414 and Ser 489. These residues were able to establish hydrogen bond, cation-π and π–π T-shaped interactions with the compound.

Regarding intramolecular interactions, the significant presence of hydrogen bonds and hydrophobic contacts are consistent with the experimental enthalpic and entropic factors, which contributed to the stabilization of DMV in the HSA's binding site, as we have experimentally demonstrated. The intermolecular interactions, shape and stereochemical complementarity suggest site I as preferential for binding. The internal ligand positioning in both sites were similar to those obtained for diapocynin in HAS^44^ and divanillin in BSA.^15^ It is worth to note that we considered a static view of the system and that conformational changes can occur through the induced fit mechanism.

Altogether, the molecular docking simulations were elucidative and corroborated experimental findings, *i.e.*, a slight DMV preference for site I. The DMV poses for site I in both crystallographic structures were similar, being internal to the cavity with the lowest energy (−8.33 kcal mol^−1^) obtained for structure 1AO6. On the other hand, in site II, the poses were internal (1E7A Cl-1) though in structure 1AO6 these were external, with the −6.82 kcal mol^−1^ obtained for structure 1E7A.

The experimental ICD spectrum of DMV matched with the theoretically calculated conformer bearing a dihedral angle of approximately −60°. Corroborant with these findings, the pose for site I (1AO6) displayed an angle of −50.9°, close to the expected. The poses for site II displayed angle values of 57.4° for the 1E7A cluster 1 and for the −51.0° to 1AO6 cluster 1, which were also close to the expected. Considering 1E7A's pose, there are two hypotheses equally plausible: (i) the first is that a local conformational adjustment can access a dihedral angle value close to the expected while still remaining in the same cluster (RMSD < 2 Å); (ii) the other is that the DMV interaction with site I leads to conformational adjustments in site II that contribute to the closing of the later, resulting in external poses that are consistent with the theoretical angle values similar to that of 1AO6 cluster 1.

## 4. Conclusions

A comprehensive study about DMV was presented, including its chemical synthesis, redox and spectroscopic properties and its interaction with albumin. As we have highlighted above, the motivation for this study was the potential application of DMV as NADPH oxidase inhibitor and/or other beneficial biological effect as have been found for the related compounds diapocynin and divanillin. Its synthesis is easily performed and not expensive, which enable the application in cell-based and *in vivo* studies. The experimental results showed that the antioxidant features of DMV were significantly improved compared to MV. DMV was also much stronger ligand of albumin compared to its precursor MV. The theoretical studies confirmed the binding of DMV at site I and II, but with a preference for site I.

## Conflicts of interest

The authors have no conflicts to declare.

## Supplementary Material

RA-009-C9RA02465D-s001
