# Supplementary material for: Methyl divanillate: redox properties and binding affinity with albumin of an antioxidant and potential NADPH oxidase inhibitor
Source: RSC Adv. 2019 Jun 26;9(35):19983–92. doi: 10.1039/c9ra02465d (PMC9065500; doi:10.1039/c9ra02465d)
Supplement: RA-009-C9RA02465D-s001 [file RA-009-C9RA02465D-s001.pdf]

Electronic Supplementary Material (ESI) for RSC Advances.
This journal is © The Royal Society of Chemistry 2019

Electronic Supplementary Information

Methyl divanillate: Redox properties and binding affinity with albumin of an antioxidant and potential NADPH oxidase inhibitor

Débora Naliati de Vasconcelos[1], Angélica Nakagawa Lima[2], Eric Allison Philot[2], Ana Lígia Scott[2], Izabelle Amorim Ferreira Boza[1], Aguinaldo Robinson de Souza[1], Nelson Henrique Morgon[3], Valdecir Farias Ximenes[1,*]

[1]Department of Chemistry, Faculty of Sciences, UNESP - São Paulo State University, 17033-360, Bauru, São Paulo, Brazil.

[2]Laboratory of Computational Biology and Bioinformatics, UFABC – Federal University of ABC, 09210-580, Santo André, SP, Brazil.

[3]Department of Physical Chemistry, Institute of Chemistry, Campinas State University (UNICAMP), 13083-861, Campinas, São Paulo, Brazil.

*Correspondence should be addressed to Valdecir Farias Ximenes.

Tel.: +55 14 3301-6088

http://orcid.org/0000-0003-2636-3080

Email: valdecir.ximenes@unesp.br

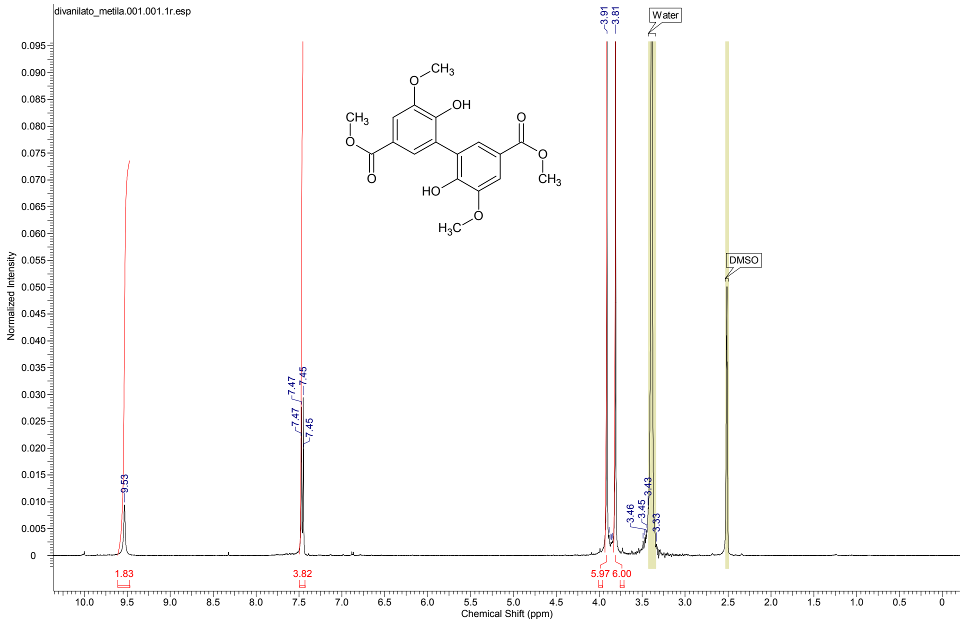

Fig. S1. ^{1}H NMR (400 MHz) spectrum of methyl divanillate. ^{1}H NMR (400 MHz) δ (ppm): (d) 9.53 (s, 2OH), 7.47 (d, 2CH-Ar, J=2.0 Hz), 7.44 (d, 2CH-Ar, J=2.0 Hz), 3.91 (s, 2 OCH3), 3.81 (s, 2 OCH3).

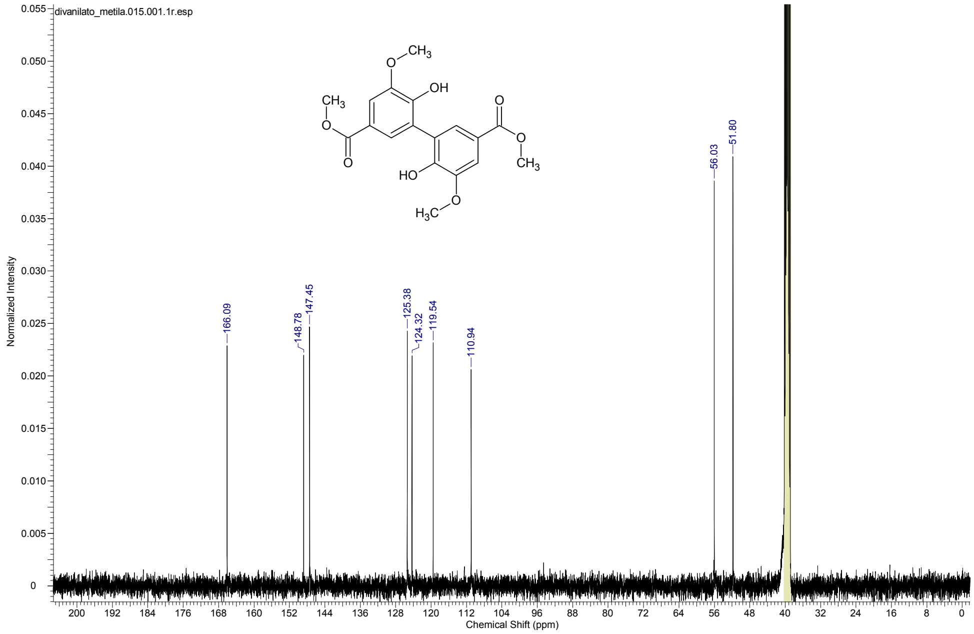

Fig. S2. ^{13}C NMR (100 MHz, DMSO-D6) d (ppm): 166.1 (2C=O), 148.8 (2C), 147.4 (2C), 125.4 (2CH), 124.3 (2C), 119.5 (2C), 110.9 (2CH), 56.0 (2OCH3), 51.8 (2OCH3).

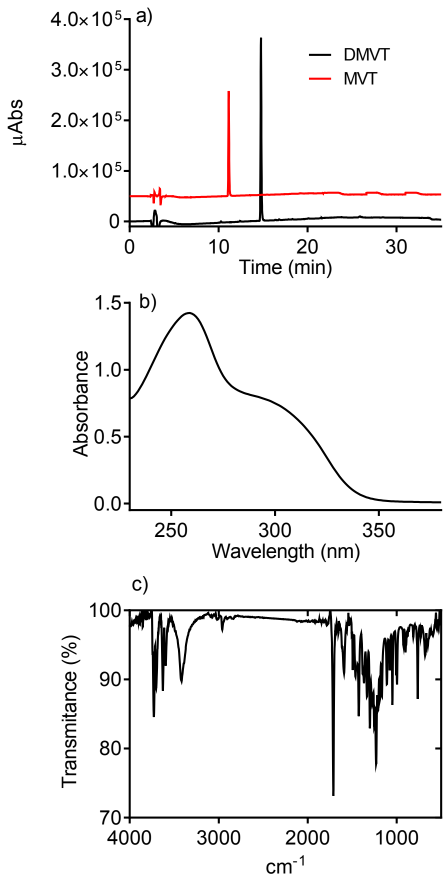

Fig. S3. a) Chromatogram of purified methyl divanillate (DMV) and its precursor methyl vanillate (MV). b) UV-Vis spectrum of DMV. c) FTIR spectrum of DMV.

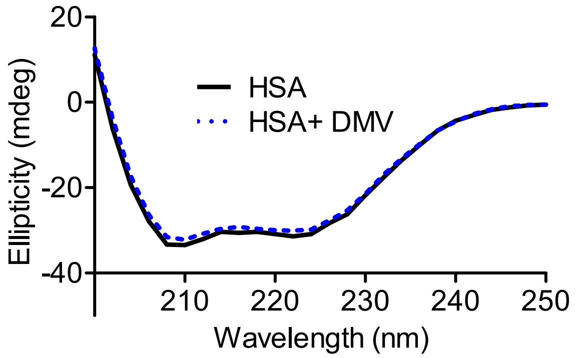

Fig. S4. Far-UV-CD spectra of HSA in the absence and presence of DMV. HSA 30 μmol L^{-1}, and DMV 30 μmol L^{-1} were incubated for 10 min and then diluted to 0.5 μmol L^{-1} before measurement.

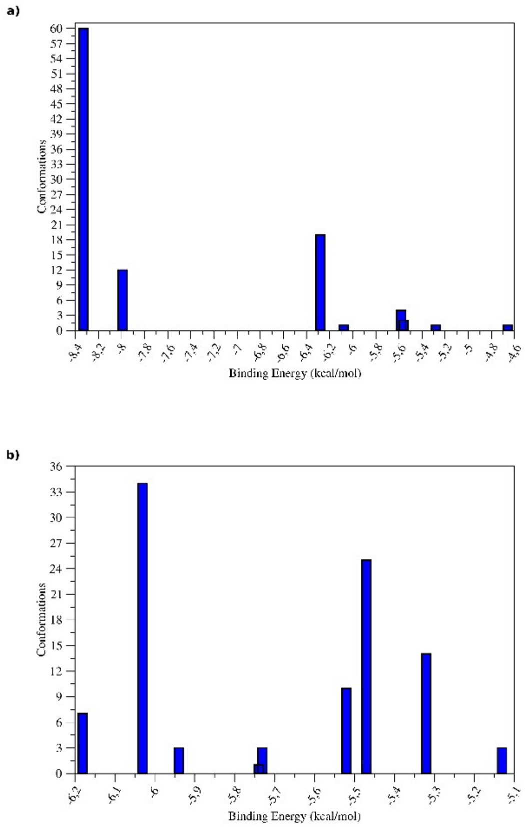

Fig. S5. Minimum energies and number of poses for each cluster of DMV obtained for the crystallographic structure 1AO6. (**a**) site I and (**b**) site II.

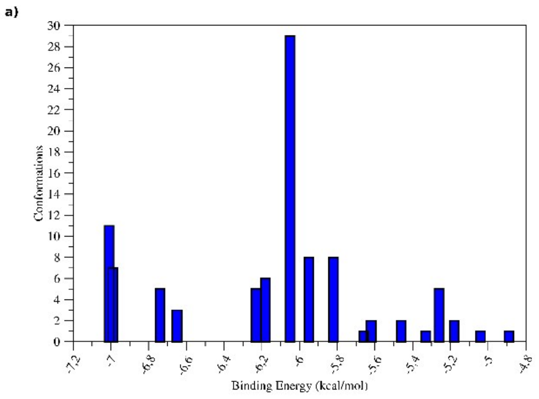

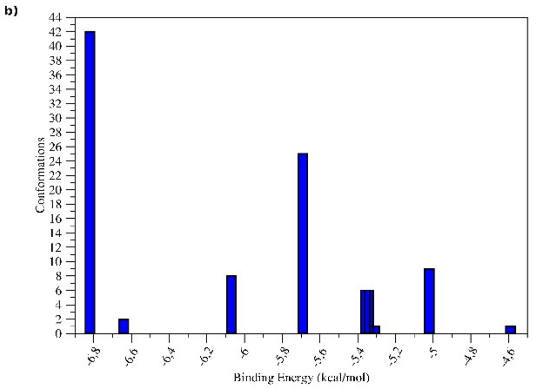

Fig. S6. Minimum energies and number of poses for each cluster of DMV obtained for the crystallographic structure 1E7A. (**a**) site I and (**b**) site II.

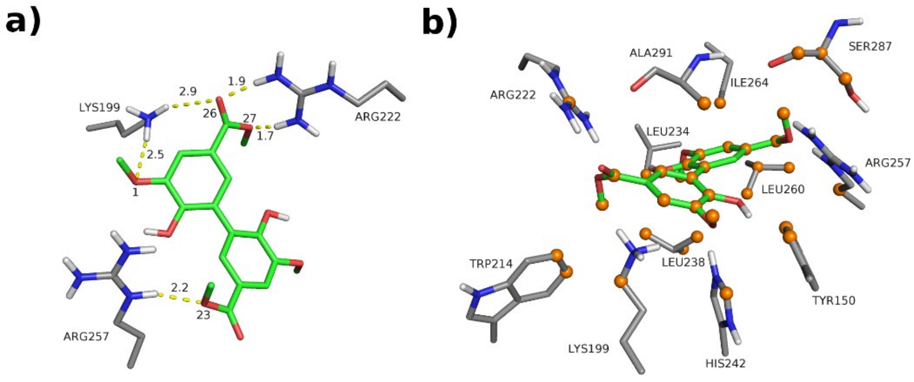

Fig. S7. Intermolecular interactions of DMV at site I of HSA (PDB 1E7A). The results represent the lower energy cluster (Cl-1). a) Hydrogen bonds; b) Hydrophobic contacts.

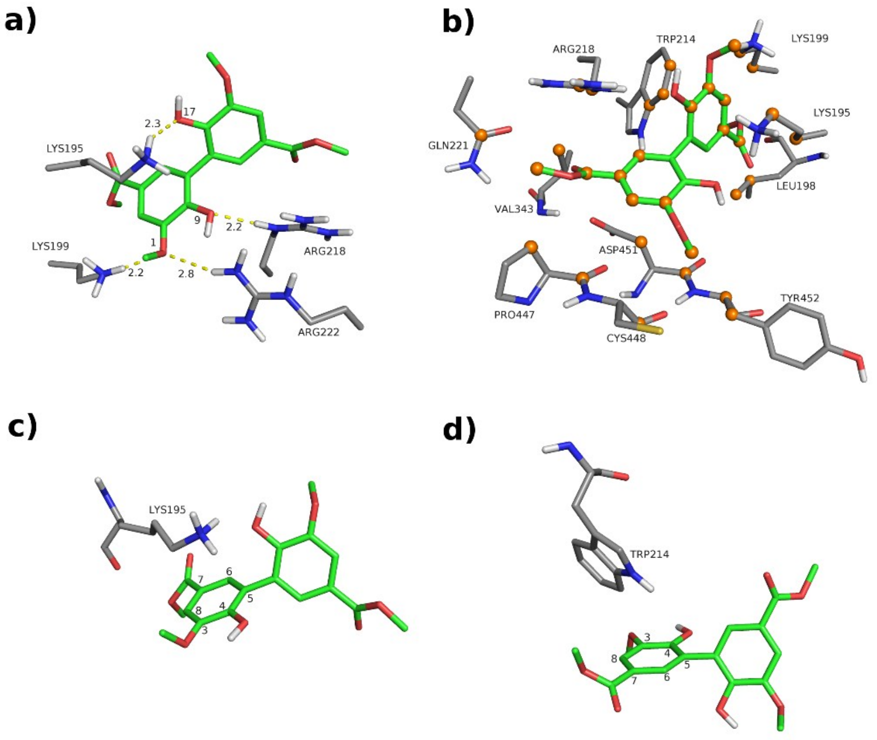

Fig. S8. Intermolecular interactions of DMV at site I of HSA (PDB 1E7A). The results represent the most populous cluster (Cl-8). a) Hydrogen bonds; b) Hydrophobic contacts; c) cation-π; d) π–π T-shaped.

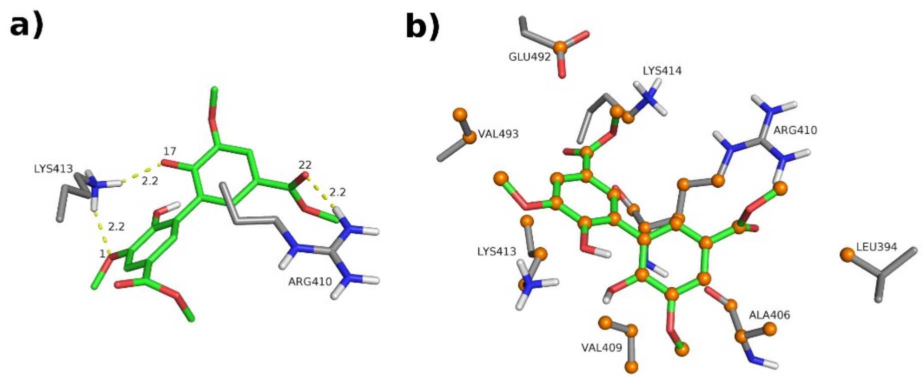

Fig. S9. Intermolecular interactions of DMV at site II of HSA (PDB 1AO6). The results represent the most populous cluster (Cl-2). a) Hydrogen bonds; b) Hydrophobic contacts.

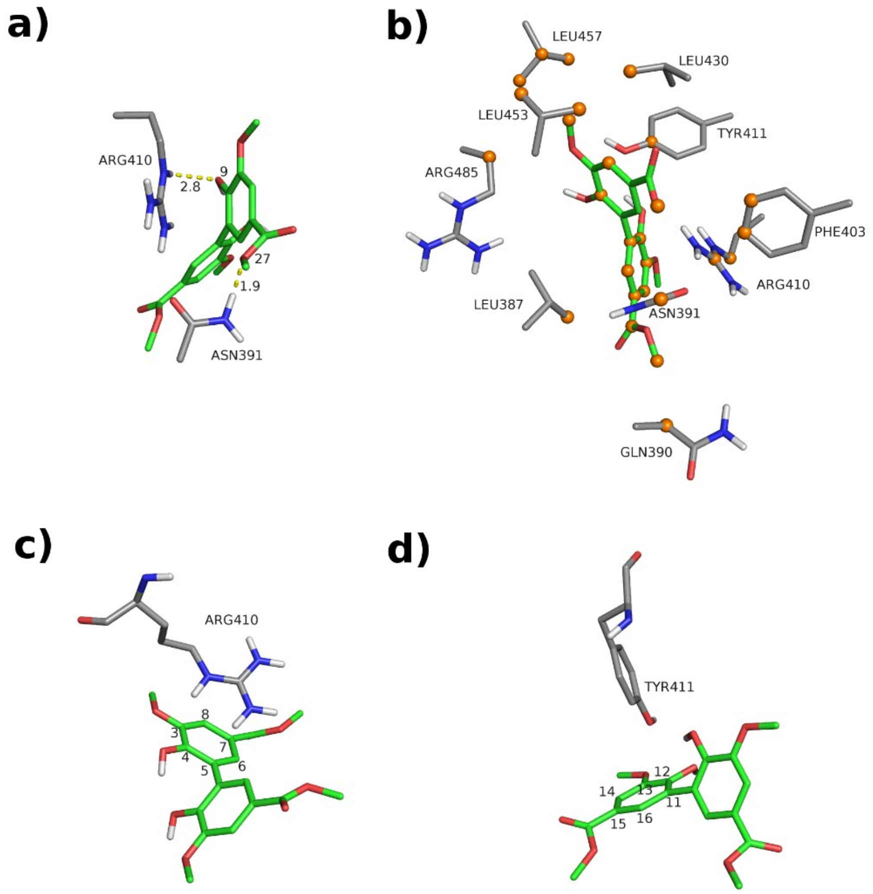

Fig. S10. Intermolecular interactions of DMV at site II of HSA (PDB 1E7A). The results represent the most populous and lower energy cluster (Cl-1). a) Hydrogen bonds; b) Hydrophobic contacts; c) cation-π; d) π–π T-shaped.

Table S1: Cytotoxicity of MV and DMV on peripheral blood neutrophil.

Compound	Cell Viability (%)* Concentration (μM) 0.1	1.0	10.0	100.0
**MV**	100,0 ± 0,0	96,4 ± 2,4	94,1 ± 1,4	82,2 ± 3,2*
**DMV**	100,0 ± 0,0	98,1 ± 1,3	95,4 ± 2,1	84,3 ± 4,1

*The results are mean and SD of triplicates.

Experimental procedure

The viability of neutrophil was assessed by using the MTT assay.[1] The neutrophils were isolated from peripheral blood of healthy donors using the double-gradient Histopaque®-1077/1119 protocol.[2] Neutrophils (1 x 10^6 cell/mL) were incubated in the absence or presence of MV and DMV in 500 μL PBS for 90 minutes at 37°C. Then, the cell suspensions were centrifuged at 4000 rpm for 5 minutes and the supernatant discarded. 500 μL of MTT (1 mg/mL) was added and the suspension incubated at 37°C for three hours, after which the medium was replaced with 100 μL of DMSO to dissolve formazan crystals. The absorbance was measured at 550 nm using a microplate reader and subtracted with the absorbance at 650 nm. The cell viability was determined as the capability of the cells to convert MTT into formazan product. Data were expressed as the percent viability compared with the control.

Table S2. Number of interactions at site I and site II.

	Site I			Site II		
	**1AO6 Cl 1**	**1E7A Cl 1**	**1E7A Cl 8**	**1AO6 Cl 1**	**1AO6 Cl 2**	**1E7A Cl 1**
**HB**	4	5	4	3	3	2
**HC**	44	33	33	47	38	25
**PIS**	---	---	---	---	---	---
**PIT**	---	---	2	---	---	1
**CPI**	---	---	1	---	---	1
**SB**	---	---	---	---	---	---

HC: hydrophobic contact | PIT: π-π T-shaped | PIS: π-π stacking | HB: hydrogen bonding

SB: salt bridge | CPI: cation-π

Table S3. Specific interactions between DMV and the amino acid residues at site I and site II.

	Site I				Site II		
**Residue**	**1AO6 Cl 1**	**1E7A Cl 1**	**1E7A Cl 8**	**Residue**	**1AO6 Cl 1**	**1AO6 Cl 2**	**1E7A Cl 1**
TYR(150)	HC	HC	---	LEU(259)	HC	---	---
LYS(195)	---	---	HB/HC/CPI	LEU(387)	---	---	HC
LEU(198)	---	---	HC	GLN(390)	---	---	HC
LYS(199)	HB/HC	HB/HC	HB/HC	ASN(391)	---	---	HB/HC
TRP(214)	HC	HC	HC/PIT	LEU(394)	---	HC	---
ARG(218)	HC	---	HB/HC	PHE(403)	---	---	HC
LEU(219)	HC	---	---	ASN(405)	HB/HC	---	---
GLN(221)	---	---	HC	ALA(406)	HC	HC	---
ARG(222)	HB	HB/HC	HB	VAL(409)	HC	HC	---
PHE(223)	HC	---	---	ARG(410)	HC	HB/HC	HB/HC/CP

							I
LEU(234)	HC	HC	---	TYR(411)	---	---	HC/PIT
LEU(238)	HC	HC	---	LYS(413)	---	HB/HC	---
HIS(242)	---	HC	---	LYS(414)	---	HC	---
ARG(257)	HB/HC	HB/HC	---	LEU(430)	---	---	HC
LEU(260)	HC	HC	---	LEU(453)	---	---	HC
ALA(261)	HC	---	---	LEU(457)	---	---	HC
ILE(264)	HC	HC	---	ARG(485)	---	---	HC
SER(287)	HC	HC	---	GLU(492)	---	HC	---
ILE(290)	HC	---	---	VAL(493)	---	HC	---
ALA(291)	HC	HC	---	LEU(529)	HC	---	---
VAL(343)	---	---	HC	LEU(544)	HC	---	---
PRO(447)	---	---	HC	LYS(545)	HB/HC	---	---
CYS(448)	---	---	HC	MET(548)	HC	---	---
ASP(451)	---	---	HC				
TYR(452)	---	---	HC				

HC: hydrophobic contact | PIT: π-π T-shaped | PIS: π-π stacking | HB: hydrogen bonding

SB: salt bridge | CPI: cation-π

References

1. FERRARI, M.; FORNASIERO, M.C., ISETTA, A. M. MTT colorimetric assay for testing macrophage cytotoxic activity in vitro. J. Immunol. Methods, v. 131, n.2, p. 165-72, 1990.
2. D. English and B. R. Andersen, Single-step separation of red blood cells. Granulocytes and mononuclear leukocytes on discontinuous density gradients of Ficoll-Hypaque, J. Immunol. Methods, 1974, 5, 249–252.
